# The Role of Systems Biology in Deciphering Asthma Heterogeneity

**DOI:** 10.3390/life12101562

**Published:** 2022-10-08

**Authors:** Mahmood Yaseen Hachim, Fatma Alqutami, Ibrahim Yaseen Hachim, Saba Al Heialy, Hauke Busch, Rifat Hamoudi, Qutayba Hamid

**Affiliations:** 1 College of Medicine, Mohammed Bin Rashid University of Medicine and Health Sciences, Dubai P.O. Box 505055, United Arab Emirates; 2 Sharjah Institute for Medical Research, College of Medicine, University of Sharjah, Sharjah P.O. Box 27272, United Arab Emirates; 3 Meakins-Christie Laboratories, McGill University, Montreal, QC H4A 3J1, Canada; 4 Medical Systems Biology Group, Institute for Experimental Dermatology, Institute for Cardiogenetics, University of Lübeck, 23562 Lübeck, Germany; 5 Division of Surgery and Interventional Science, University College London, London KT1 2EE, UK

**Keywords:** asthma, systems biology, multi-omics

## Abstract

Asthma is one of the most common and lifelong and chronic inflammatory diseases characterized by inflammation, bronchial hyperresponsiveness, and airway obstruction episodes. It is a heterogeneous disease of varying and overlapping phenotypes with many confounding factors playing a role in disease susceptibility and management. Such multifactorial disorders will benefit from using systems biology as a strategy to elucidate molecular insights from complex, quantitative, massive clinical, and biological data that will help to understand the underlying disease mechanism, early detection, and treatment planning. Systems biology is an approach that uses the comprehensive understanding of living systems through bioinformatics, mathematical, and computational techniques to model diverse high-throughput molecular, cellular, and the physiologic profiling of healthy and diseased populations to define biological processes. The use of systems biology has helped understand and enrich our knowledge of asthma heterogeneity and molecular basis; however, such methods have their limitations. The translational benefits of these studies are few, and it is recommended to reanalyze the different studies and omics in conjugation with one another which may help understand the reasons for this variation and help overcome the limitations of understanding the heterogeneity in asthma pathology. In this review, we aim to show the different factors that play a role in asthma heterogeneity and how systems biology may aid in understanding and deciphering the molecular basis of asthma.

## 1. Introduction

Asthma is a common and lifelong chronic inflammatory disease characterized by inflammation, bronchial hyperresponsiveness, and airway obstruction episodes [1,2]. In 2019, the Lancet reported 262 million cases of asthma, with 0.461 million deaths [3].

The complexity of the disease, the unexplained heterogeneity in clinical presentation, and the involvement of various environmental and genetic determinants in asthma affect our understanding of the mechanisms involved in the disease process and the interaction between the different determinants [4,5,6]. There is a compelling picture of unmet needs in the prevention, treatment, and curing of asthma that needs to be addressed in a more systematic and collaborative approach [7].

Recently, systems biology was introduced as a possible approach to addressing the complexity of the many underlying disease mechanisms through the analysis of complex, quantitative, massive clinical and biological data [8]. This approach uses the comprehensive understanding of living systems through bioinformatics, mathematical, and computational techniques to model diverse high-throughput molecular, cellular, and physiologic profiling of healthy and diseased populations to define biological processes [9]. Such an approach was shown to have great potential in deciphering the complexity and heterogeneity of allergic diseases, including asthma, by understanding the variable interaction between environmental perturbations and genetic predisposition. In this review, we aimed to review the variable factors involved in asthma etiology, its complex phenotypes, and the role of systems biology in providing answers for asthma research.

## 2. Etiology of Asthma

### 2.1. Genetic Factors

There was an important progression in the discovery of genetic factors involved in asthma susceptibility. Various studies identified approximately 18 genomic regions, and 100 genes were found to be linked with asthma and allergic diseases in different populations [10]. Moreover, these genetic variations were found to be responsible for 50% of asthma development risk, especially in childhood-onset asthma [11]. Many of the identified asthma susceptibility risk loci were found to be associated with immune cell function. Additionally, these are shared with other autoimmune and inflammatory diseases such as Th2 cell homeostasis, activation genes, and innate immunity and regulatory genes [12,13]. Recently, bioinformatics analyses have prioritized candidate causal genes at 52 loci carrying asthma-associated variants enriched in regions of open chromatin in immune cells [14]. However, asthma heritability is incomplete and not fully understood yet.

### 2.2. Environmental and Toxicogenomic Factors

There have been increased concerns about what and how different environmental exposures might trigger asthma exacerbations and how they play a role in increasing the rate of asthma incidences [15]. These factors include drugs, toxins, chemicals, and infections, although their role and mechanisms in asthma development and exacerbations are not yet fully understood [16]. Other environmental factors include allergens, occupational sensitizers, and outdoor and indoor pollution [17,18]. While air pollution’s adverse effects on lung function are well documented in asthmatics, there is still a debate on how these air particles can trigger asthma [19,20]. Food is an external or modulated asthma initiator, with children having food allergies being more prone to food-induced episodes of asthma that can result in anaphylaxis [21]. Drug-induced asthma is another well-defined and relatively common and often-under-diagnosed asthma phenotype, especially aspirin-induced asthma [22,23].

### 2.3. The Immune System in Asthma

**Figure 1:**
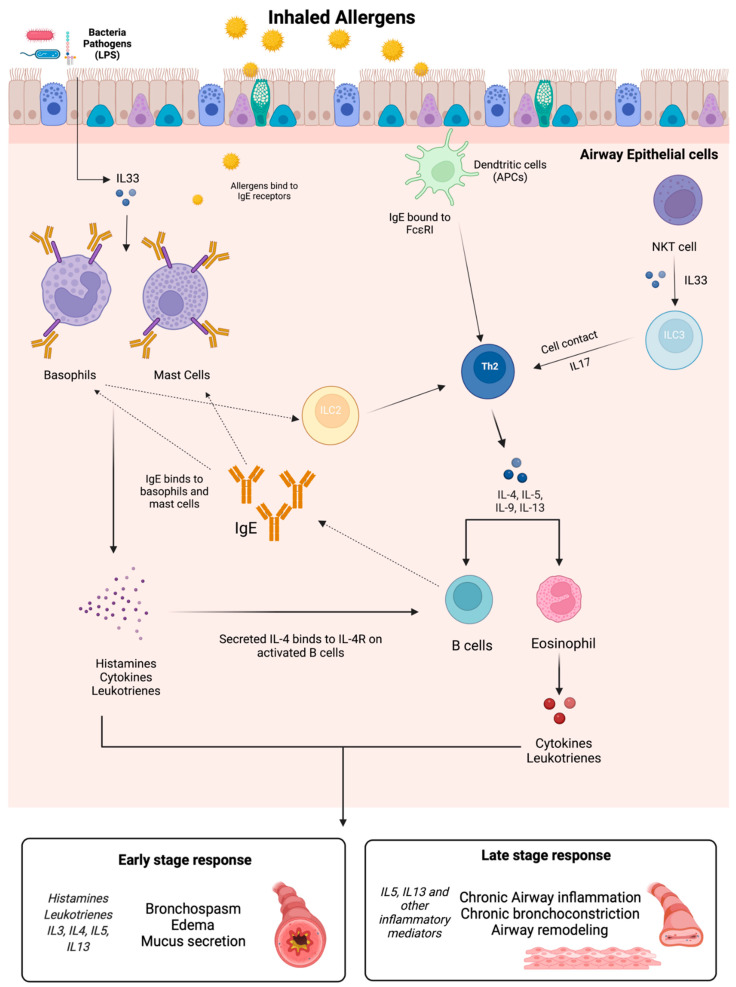
IgE and its mediators play a role in both early stage and late-stage responses in asthma upon exposure to allergens. These responses rely on multiple cells, including dendritic cells, ILC cells, Th2 cells, B cells, eosinophils, basophils, and mast cells. Such cells release many mediators, including histamines, leukotrienes, and various cytokines, such as IL3, IL4, IL5, and IL13. The types of cells and mediators present during an asthma episode are dependent on the subtype, and not all cells illustrated above might be involved.

The pathophysiology of asthma relies on multiple types of cells, mediators, and pathways prompted by molecular and cellular events that lead to the development and progression of asthma (Figure 1). Cells that play a role in asthma include neutrophils, dendritic cells, basophils, and eosinophils, each present at different stages of asthma progression.

## 3. Diagnosis and Management

The diagnosis of asthma is not as clear-cut in comparison to other diseases due to its reliance on the clinical judgment of patient history, symptoms, measures of airflow obstruction, and respiratory inflammation. The absence of a single diagnostic test or a series of tests to confirm asthma or accurately label its phenotype has highlighted the need to identify reliable biomarkers to accurately diagnose asthma and define the underlying pathophysiological pathways of different clinical asthma presentations [24,25].

**Figure 2:**
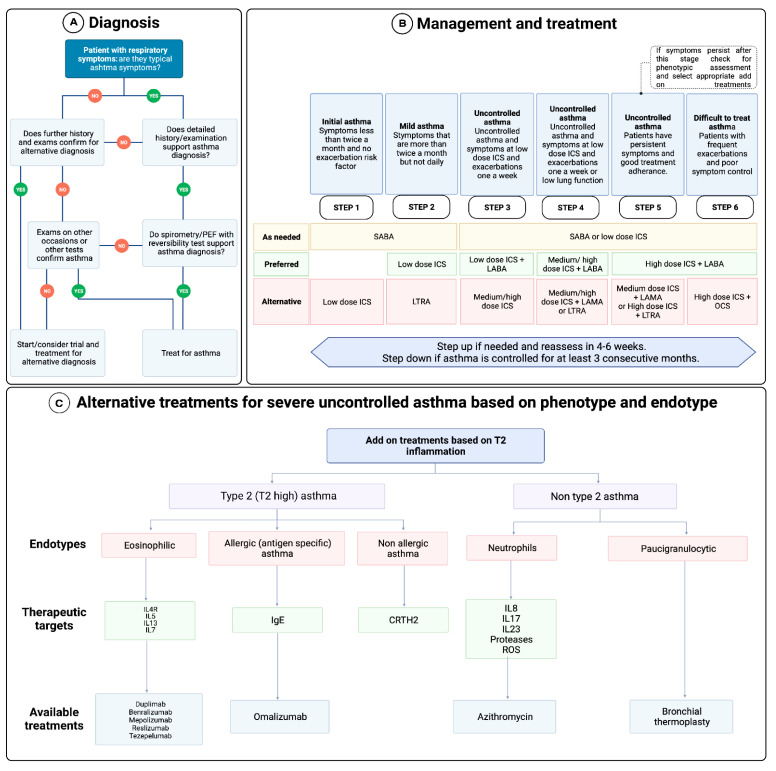
Asthma diagnosis and management involve a multi-step plan that begins with (**A**) proper diagnosis for the disease followed by (**B**) the 6-step plan that aims to control symptoms, reduce exacerbations, and any adverse event that is a product of the therapeutics used. (**C**) Alternative treatments, including biologics, are administered to patients whose asthma is uncontrollable by standard treatment methods and is dependent on endotyping and clinical biomarkers. Omalizumab is the first biological treatment for allergic asthma and targets IgE.

In terms of asthma management, managing both short and long-term respiratory and other health outcomes is a must [26]. The primary endpoints include the control of symptoms, fewer exacerbations, improved lung function, and the minimizing of adverse events (A.E.s) caused by the use of therapeutics (Figure 2) [27]. In 90–95% of patients with asthma, symptoms are well controlled with inhaled therapy used to prevent exacerbations, while a minority of patients who fail to respond are categorized as having severe asthma [28].

Inhaled corticosteroids (ICS) are the most common asthma treatment method, with other quick-relief and rescue medicines used in an adjustable severity-based six-step management plan [29,30]. Another cornerstone in asthma management is controlling environmental factors, including allergen mitigation and intervention [30].

A new approach was proposed with the increasing understanding of molecular pathways in severe asthma phenotypes. This includes the use of biological therapies that target components that were found to drive asthma symptoms, which have been investigated and approved (Figure 2C). Some of these biologics include monoclonal antibodies such as Omalizumab that target IgE or IL5 targets such as Mepolizumab, Reslizumab, and Benralizumab [27]. Patients with childhood asthma have been found to respond better to IgE-targeted therapeutics, while adult-onset asthma patients respond better to anti-IL5 antibodies and therapies [31]. However, choosing the necessary biological treatment depends on the asthma endotype, clinical biomarkers, and patient-focused aspects [32].

## 4. Systems Biology

**Figure 3:**
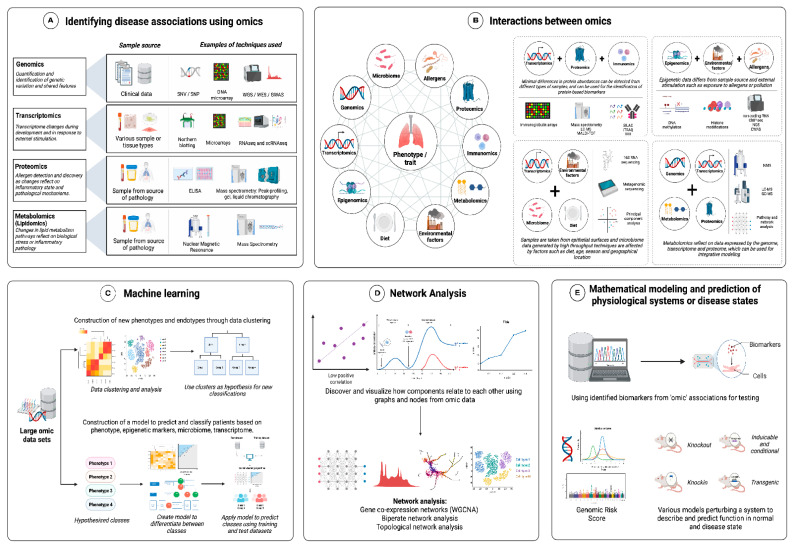
The application of systems biology involves (**A**) identifying the disease associations using various techniques from various omics such as genomics and transcriptomics. (**B**) Studying the interactions between the various omics, such as metagenomic sequencing, involves transcriptomics and the microbiome, and is affected by environmental factors; (**C**) Machine learning uses the data generated from large omics datasets to create new sets of classification or predictive models. (**D**) Network analysis uses omics data to discover and visualize the relationship between various disease components, which is then (**E**) used to create a mathematical model to identify biomarkers and an in-silico model for studying disease mechanisms.

Systems biology refers to the systemic investigation of living systems through bioinformatics, mathematical, and computational techniques to model diverse high-throughput molecular, cellular, and physiologic profiling of healthy and diseased populations to define biological processes (Figure 3) [9]. This process involves using various omics data such as genome, epigenome, transcriptome, proteome, metabolome, and microbiome and incorporating high-throughput methods such as machine learning to explore the heterogeneity of the diseases, as well as to correlate, discriminate, and predict between disease phenotypes.

### 4.1. Why Systems Biology Approach Is Needed in Asthma?

Asthma and other multifactorial diseases represent an ideal candidate for systems biology approaches. In the context of such diseases, approaching the condition from a system’s biology perspective might help extract new insights from complex, quantitative, massive clinical, and biological data to understand the underlying mechanisms of diseases and aid early detection and treatment [8,33] through identifying the biomolecular networks driving the disease and the construction of regulatory networks and biological pathway models [33,34,35].

Currently, severe asthma patients have no effective treatment to control the disability and mortality of asthma, and they may not respond to steroids due to the relative glucocorticoid insensitivity [36,37]. Therefore, there is a need for a continuous effort to refine the current phenotypes to improve patients’ classifications, allowing for more precise and personalized therapeutic options [38].

In addition, there are no asthma risk assessment tools for food, drugs, occupational and household chemicals. The use of systems biology and multi-omics to analyze the response of all genes to such chemical exposures can be examined to understand the potential hazards of such irritants and toxicants [39]. It has been shown that by using the in silico gene expression data and linking it to toxicogenomic data, differentially expressed genes can be used to explain and predict the effect of toxicity on an asthmatic epithelium [40].

### 4.2. Multi-Omics

Multi-omics approaches involve the interaction between different omics to decipher the mechanisms of asthma. There has been an increase in evidence in the role of epigenetic and environmental regulation on asthma phenotypes. Thus, the use of epigenetic-modifying tools such as histone modification techniques to target the various hallmarks of asthma is of interest as it aids in developing novel therapeutics [41]. Additionally, the use of multi-omics in tandem with genome-wide association studies (GWAS) through an upper airway epithelial cell (AEC) culture model to assess transcriptional and epigenetic responses to rhinovirus (R.V.) reveal a specific genetic mechanism found at the risk locus of childhood-onset asthma [42]. Such revelations provide context-specific functional annotations to variants that were discovered in the GWASs of asthma [42].

However, integrating data omics requires specific tools and pipelines; an example is the Merged Affinity Network Association Clustering (MANAclust). It is a pipeline that integrates categorical and numeric data that span clinical and multi-omic profiles for unsupervised clustering. This clinical and molecular distinct clustering is then used to identify disease subsets to a phenotypic asthma cohort, including heterogeneous groups and subsets of healthy controls and asthmatic subjects [43].

## 5. Systems Biology Findings and Applications in Asthma

### 5.1. Asthma Classification, including Phenotyping and Genotyping

In asthma, several sub-phenotypes of asthma have been implemented, with the most common phenotype being allergic asthma and patients showing exaggerated responses to nonharmful agents [44]. A newer approach to classifying asthma is cellular phenotyping, where the type and quantity of inflammatory cells in the airway are used to guide the selection of ideal biological treatments [45]. Asthma can be broadly classified as eosinophilic or non-eosinophilic (NEA) based on airway or peripheral blood cellular profiles [46]. For example, differentiating between non-eosinophilic and eosinophilic asthma is essential in terms of using corticosteroids, the mainstay therapy in asthma [47] as NEA is generally poorly responsive to corticosteroid treatment, which could potentially worsen the disease [46].

Molecular phenotyping involves quantitating proteins, posttranslational modifications, metabolites, and nucleic acids using high-throughput analytics [47]. Among the various omics, the bronchial epithelium transcriptomics-driven phenotyping of asthmatic patients revealed the potential to discover gene expression profiles characteristic of asthma [48]. Furthermore, bronchial epithelium transcriptomics can identify different molecular mechanisms underlying divergent asthmatic phenotypes and have the power to identify novel clinically efficient biomarkers [49,50]. An example would be using transcriptomic data to determine the differentially expressed gene JMJD2B/KDM4B in asthmatic airway fibroblasts, expressed upon IL-13 exposure [41].

Recently, a combined approach that includes genomic, molecular biology, and a comprehensive phenotyping approach called phenomics was introduced to identify disease subtypes, including asthma [51]. Such an approach might be essential to improve our characterization of severe asthma heterogeneity by implementing evidence-based criteria. Indeed, this might help in the adoption of more individualized therapeutic options for asthmatic patients, including patients with the severe form [51]. Moreover, the extraction of information from the electronic health record (EHR) systems, including clinical history, risk factors, and history of exposure to various materials and allergens and combining them with information obtained from biospecimen collection that involves genetic variants led to the development of new types of studies called phenome-wide association studies (PheWASs) [52]. In addition to its role in exploring novel asthma-related genetic variants and risk loci, it is also essential to identify risk factors and comorbidities [52]. In addition, PheWAS was also found to play a role in identifying new therapeutic candidates and predicting adverse drug effects. For example, one report highlighted asthma as a possible adverse event when using PNPLA3 inhibitors to manage liver diseases [53].

### 5.2. Systems Biology Enables the Identification of Biomarkers

Identifying novel markers that can help in a patient’s classification and clinical outcome prediction and response to therapy was and will remain the ultimate goal for many research projects [54,55]. These markers should have precise specifications and criteria such as reliability, ease of collection, and measurement—and not be invasive. For example, using reliable biomarkers to predict asthma patients’ response to steroid therapy early in the disease is particularly important and necessary to achieve optimal response and avoid undesirable side effects [56].

Testing biomarkers is not routinely requested for severe asthma patients, and the most extensively studied biomarkers are those that are related to the T2 phenotype [57,58]. For biomarkers to be ideal, they should be reliably quantifiable, easy to obtain, cost-effective, and can be produced in different clinical settings [59].

PBMCs containing the significant sources of allergic response mediators in asthma can serve as an excellent alternative to the costly and challenging method of obtaining airway samples in severe asthma [60]. Furthermore, the integrative phenotype–genotype approach is a novel, simple, and powerful tool for identifying clinically relevant potential biomarkers. This approach was been recently used to identify clinically essential biomarkers in complex and heterogeneous diseases such as diabetes [61]. Several genes such as SERPINE1, GPRC5A, SFN, ABCA1, MKI67, and RRM2 have been found to be downregulated in severe uncontrolled asthma using PBMCs and, therefore, potential biomarkers [62]. These biomarkers were initially identified from a list of genes using in silico techniques such as GWAS and methylomes and were further validated using in vitro studies [62].

### 5.3. Genomics and Their Role and Applications in Asthma

The genetic component is a significant factor contributing to asthma etiology, with estimated heritability varying from 35% to 95% [63]. For that reason, significant efforts were made to identify the genetic map associated with asthma and led to the discovery of potential contributory genes [64]. Recently, genome-wide association analyses (GWAS) analysis led to the identification of genetic loci that were shown to be found across many allergic phenotypes, including hay fever/allergic rhinitis, atopic dermatitis, food allergy and asthma [33]. Some of those genes were related to the human leukocyte antigen (HLA) locus, including HLA-DQ/DRB1, HLA-DQA1/2, and HLA-B/C [65]. Others were linked to immune function, including thymic stromal lymphopoietin (TSLP), IL13, IL4, and IL33, which were shown to be associated with the promotion of Th2-type cytokine synthesis and T2 immune response [66]. While some of the discovered genes were linked to asthma pathophysiology, the role of other genes such as WDR36 and CLEC16A is still uncertain and needs further investigation [33]. Other promising asthma candidate genes include the beta-2 adrenergic receptor gene (ADRB2). The role of this gene in the risk of asthma is further supported by the efficacy of inhaled β2-adrenergic receptor agonists in the management of asthma [67]. Other frequently investigated genes include FCER1B (MS4A2), which encode the beta subunit of the high-affinity IgE receptor, TNFA, a proinflammatory cytokine, and the CD14 surface antigen gene [64].

Some genes were linked to specific asthma phenotypes, including ORMDL3/GSDMB/LRRC3C (17q21.1), which is linked with childhood asthma [68]. Other genes showed ethnicity-specific patterns, including PYHIN1, that were associated with asthma but only in persons of African ancestry [69].

### 5.4. Transcriptomics and Its Applications in Asthma

This approach includes various types of RNA transcripts profiling, including mRNA, noncoding RNA (ncRNA), and microRNA. Using DNA microarrays and RNA-sequencing (RNA-Seq) in the last decade, many genes and pathways were discovered, improving our understanding of asthma’s molecular mechanisms [70]. Variable cells were suitable for transcriptomic studies in asthma, including whole blood cells, peripheral blood mononuclear cells, and lymphoblastoid B cells. Other transcriptomic analysis sources are the sputum and other epithelial cells obtained from nasal epithelia and bronchial brushing. Bronchoalveolar lavage (BAL) was another source of cells as it might reflect the internal environment present in the lower respiratory tract [71].

White blood cell differential expression analysis between healthy individuals, controlled asthma, and therapy-resistant severe asthma revealed the increment in TAS2R pathways in severe asthma [72]. Another study also revealed a reduction in glucocorticoid receptor signaling and the upregulation of mitogen-activated protein kinase and JNK cascade activity in children with severe asthma compared with those with controlled ones [48]. PBMCs gene expression profiling in asthmatic children revealed a strong association between the high neutrophil count and inadequate treatment control in Th1/Th17-mediated asthma [73]. Our lab also identified ten genes involved in cell cycle and proliferation, including ABCA1, GPRC5A, KRT8, SFN, TOP2A, SERPINE1, ANLN, MKI67, NEK2, and RRM2 to derange in the bronchial epithelium and fibroblasts of severe asthmatic patients compared to healthy individuals. SERPINE1, GPRC5A, SFN, ABCA1, MKI67, and RRM2 were also downregulated in the PBMCs of severely uncontrolled asthmatic patients [74].

Other studies across multiple tissue types revealed the differential expression of genes and pathways involved in inflammatory and repair responses and epithelial integrity, in addition to genes involved in innate and adaptive immunity [33]. Some reports also highlighted a strong association between distinct transcriptomic profiles and inflammatory asthma subtypes. For example, elevated periostin (POSTN), CLC, CLCA1, SERPINB2, DNASE1L3, and CPA3 were found to be associated with eosinophilic or T2-driven airway inflammation; in contrast, the expression of DEFB4B, CXCR2, IL1B, ALPL, and other chemokines were linked to neutrophilic or Th17-linked inflammation [75].

### 5.5. Microbiome and Asthma

While many multi-omic studies focus on genomic and transcriptomic approaches, there has been an increase in using omics technologies in assessing the effect of the environment on the disease [76]. Current research data suggest that environmental factors such as microbiome and diet interact with genetic and epigenetic factors, leading to disturbance in immune development and the balance of vital inflammatory pathways.

Several studies link changes in the gut microbiome and early life risk factors for diseases such as asthma, though they have focused on one risk factor impacting individuals rather than populations [77,78]. Therefore, a systems biology approach via microbiome analysis can provide an understanding of asthma and its complexity and improve patient classification methods, status monitoring, and therapeutic choices [79].

Microbial products are one of the candidates for primary asthma prevention as exposure to them or their products results in the reprogramming of innate immunity as well as protection against allergies and asthma development in children [80]. An example would be neutrophilic asthma, which involves airway microbiota [81]. Refractory neutrophilic asthma has been associated with specific microbial signatures using microbiome data alongside host multi-omic data [79]. Such signatures include predominant pathogenetic bacteria, including Gammaproteobacteria, especially species from Haemophilus and Moraxella [79].

Chronic cough caused by persistent bacterial bronchitis (PBB) has been previously misdiagnosed as asthma, and patients have incorrectly prescribed asthma therapies, resulting in an escalation of their symptoms. Previously PBB consisted of a single diagnosis, but it is now divided into several subtypes (Table 1), indicating the utmost importance of the need for extended endotyping. Furthermore, such extensive subtyping signals a move towards omics and systems biology due to the similar responses in phenotypes across different diseases and conditions—such is the case of neutrophilic airway disease [82].

### 5.6. Metabolomics and Breathomics in Asthma Research

Metabolomics is one of the systems biology studies that usually involve a comprehensive measurement of metabolites, including quantifying and assessing low molecular weight compounds in various biological samples [83]. The wide range of biospecimens that can be used in metabolism, including several other non-invasive techniques such as plasma, serum, and exhaled breath, highlighted the potential benefits of using such techniques in asthma research [84]. Such an approach might be essential for discovering potential pathogenic pathways involved in asthma pathogenesis and novel biomarkers discovery, in addition to therapy response prediction [85]. A recent study using metabolomics identified a distinct metabolic profile in asthma that could precisely differentiate between mild, moderate, and severe forms of asthma [85]. Their report highlighted a modest systemic metabolic shift in a severity-dependent manner in asthmatic patients [85]. While exogenous metabolites, including elevated dietary lipids, represent a primary metabolic shift in mild asthma, the moderate and severe asthmatic patients showed a distinct, abundant metabolite including sphingosine-1-phosphate (S1P), OEA as well as N-palmitoyltaurine [85]. Other reports also showed a distinct metabolic profile of severe asthma compared to mild-to-moderate asthma, including amino acids metabolism, which usually showed elevation in β-alanine or lysine levels [86,87]. Several lipid mediators were also shown to correlate positively with asthma severity, including sphingolipids, free fatty acids, and eicosanoids (LTE4) [85,88]. Another report also highlighted serum glycerophospholipid metabolic profile as a marker that was able to differentiate between eosinophilic and non-eosinophilic asthma [89]. Another important application of metabolomics in asthma is the prediction of resistance to steroid therapy. Higher levels of linoleic acid metabolite were found to be responsible for steroid resistance through the NF-κB pathway [90].

Recently, breathomics, an evolving branch of metabolomics that includes the analysis of exhaled breath condensate, was found to be beneficial in the characterization of asthmatic subjects, including asthma endotyping. A novel study revealed that the use of 15 volatile organic compounds in exhaled breath samples was able to stratify asthmatic patients into eosinophilic (>2% sputum eosinophilia) endotype and neutrophilic (≥40% sputum neutrophilia) endotype [91]. Moreover, the evaluation of volatile organic compounds (VOCs) might predict the body’s chemistry changes and be found to be able to monitor the pharmacodynamics and pharmacokinetics of many drugs used in asthma management [92,93]. For example, one report highlighted that exhaled breath volatile organic compound levels could predict the responsiveness of steroids in mild/moderate asthma with accuracy greater than sputum eosinophils and FeNO [94].

### 5.7. Epigenome, Environment, and Asthma

Epigenomics includes heritable biochemical modifications that cause changes in gene expression without changes in the DNA sequence. Those modifications include DNA methylation, histone modifications, and microRNAs (miRNAs) [95]. Epigenetic changes were proposed to play a role in the pathogenesis of asthma. For example, one report showed that exposure to a high methyl donor diet in utero might cause an increment in airway inflammation and an increase in the serum IgE that might facilitate the T2 phenotype in lymphocytes as well as the hypermethylation of Runx [96].

A methylation array-based report performed on bronchial brushings and mucosal biopsy revealed a novel methylation mark that can differentiate between asthmatic and atopic individuals compared to healthy controls [97]. Moreover, other epigenetic mechanisms were shown to be involved in the regulation of cytokines and transcription factors involved in T cell differentiation [98]. One report highlighted the role of the global DNA demethylation agent (5-AZA) in allergic airway disease by preventing Th2 skewing, and Th1/Th2 rebalance [99].

A study focused on genome-wide histone modification profiles in a group of cells, including naive, TH1, and TH2 cells obtained from the blood of asthmatic patients compared to healthy individuals which identified enhancers associated with the TH2 memory cell that showed distinct histone H3 Lys4 dimethyl (H3K4Me2) enrichment, which differs according to the status of asthma [100].

Reports also highlighted the presence of up-and-down-regulated miRNAs in asthmatic patients compared to healthy individuals. Those miRs were found to be associated with epithelium development, homeostasis, and inflammatory pathways [101]. This includes let-7f, miR-181c, and miR-487b, which were found to be elevated in mild asthma patients compared to normal. In contrast, miR-203 was shown to be reduced in those patients compared to the healthy control [101]. In addition, some miRNAs were also found to be associated with allergy risk. For example, both miR-155 and miR-146 were found to play an essential role in T cells skewed towards Th2 versus Th1/Th17 [102].

Epigenomic changes may also appear as a response to interaction with endogenous or exogenous environment factors [103]. Moreover, reports also showed evidence of a possible role of environment-gene interactions in determining asthmatic phenotypes [104]. For example, one report showed an increment in protocadherin-20 (PCDH20) methylation in asthmatic patients who smoke. This change was evident even after environmental factors were adjusted [105]. Evidence also emerged about a possible synergistic interaction between genetic and epigenetic variations in the modulation of gene expression. Examples include DNA methylation interaction with SNPs variations in T-helper 2 pathway, interleukin-4 receptor gene [106].

## 6. Limitations of Systems Biology in Asthma

### 6.1. Limitations

In recent years, there has been an increase in the use of a systems biology approach to asthma and allergy, which has provided great results [33]. However, although they have provided extraordinary details and enriched our knowledge about asthma heterogeneity and its molecular basis, these techniques gave no conclusive and even contradictory results in asthma [107,108].

While transcriptomic data and transcriptomics provide more dynamic details that are essential in understanding active heterogeneous diseases such as asthma and can help in its “prediction” and “diagnosis” [109], in several well-designed transcriptomic studies, there was no link between the identified transcriptomic data and clinical findings, indicating missing biological relevance which can be identified if more comprehensive designs and data analysis are tried [70,110].

Asthma genome-wide SNP association studies is a hypothesis-free approach that relies on data gathering, analysis, and interpretation to identify susceptibility genes; however, mutation analysis can only explain 2.5% of the variation and is inconsistent between ethnic groups [111,112].

Many publicly available transcriptomic datasets were made through arrays; however, microarray technology can carry noise and errors due to intra-cellular heterogeneity, which in turn can shift gene expression readings based on the examined conditions [113,114,115]. A shift can be seen in the extreme method-to-method variation in the results of the significant differentially expressed genes (DEGs) in many studies [116]. Such experimental process variability can mask the investigated biological effects, affecting the proper identification of truly DEGs from genes that are equally expressed between disease states and controls [117,118].

This relatively expensive approach can be more informative if a large number of samples are combined to extract meaningful information by using a large number of datasets available in public databases [119]. However, such an approach has not yet been performed in a broader range of patients, so these omics studies have only provided a partial view of the disease due to their focus on specific subtypes rather than the disease as a whole [76,120]. For example, “Systems Pharmacology Approach to Uncontrolled Pediatric Asthma” is a study that uses systems-wide omics in a layered manner to identify the pathophysiological mechanisms, specifically in moderate-to-severe uncontrolled pediatric asthma, rather than assessing across populations [121].

The translational benefit of asthma genomic studies is sparse, and known asthma susceptibility genes can marginally increase the disease risk due to the genetic and phenotypic heterogeneity of asthma [122]. Thus, a deeper understanding of the novel regulatory mechanisms that might affect the function of susceptibility genes might overcome this limitation in understanding the heterogeneous manifestations of asthma pathology [123].

While omics, data science, and systems biology have better enhanced our understanding of the molecular mechanisms in asthma and allergic diseases pathology, the use of omics testing in these diseases is not considered the standard of care. Several factors must be addressed before the use of these technologies can be effectively implemented in clinical practice. An example would be integrating these systems with the use of clinical decision support systems within electronic medical records for this technology to be used in clinical settings [76]. However, combining and integrating systems medicine and omics data into clinical practice may enable a more precise and personalized way of effectively managing asthma [33].

### 6.2. Confounding Factors Are a Limitation of Systems Biology

Another limitation that blocks the proper utilization of transcriptomic datasets is the countless elusive experimental factors called ‘confounding factors’ [124]. These factors can be biological or non-biological, and their identification is essential for accurate transcriptomic analysis; however, they are hidden and difficult to detect [124,125,126]. Biologists often accept or ignore such factors, considering them an inevitable part of any biological experiment. However, if these factors are considered confounding, they could result in a systematic bias that can affect the results [127]. An example of the effect of systemic bias would be the inconsistency of epidemiological, experimental, or clinical findings that are due to local and regional variations in both the environment and population genetics [128].

### 6.3. Data Integration Strategies, Such as Machine Learning, Dimension Reduction, Clustering, and Network Analysis in Asthma

The integration of multi-omics data is still considered the main challenge in fulfilling the potential of comprehensive systems biology to create a workflow capable of simultaneously modeling and investigating the interactions between multiple-omics data blocks [129]. Multi-omics analyses considering genetic, epigenetic, and functional data should be used to create an effective systems biology-based approach to developing accurate risk profiles for disease [130]. Machine learning approaches explore asthma heterogeneity to determine endotypes that correlate with the sub-phenotypes of asthma and allergy using mathematical models on genomic, transcriptomic, and proteomic data [33]. There is a great potential to maximize a single omics approach utilization by integrating them with other omics [131]. For example, recently, merged affinity network association clustering (MANAclust), a coding-free automated pipeline enabling the integration of categorical and numeric data spanning clinical and multi-omic profiles for unsupervised clustering to identify disease subsets was applied to a clinically and multi-omically phenotyped asthma cohort and was able to identify clinically and molecularly distinct clusters, including heterogeneous groups of “healthy controls” and viral and allergy-driven subsets of asthmatic subjects [43]. Additionally, an exposome study profiling joint lifestyle and environmental factors in integrated lung function in adults with asthma using a cluster-based approach revealed significant associations that did not have any direct meaning when considered independently [132]. Another promising approach is using “Development of ALLergy” that integrates the epidemiologic, clinical, and in vivo and in vitro models of 44,010 participants and 160 cohort follow-ups between pregnancy and age 20 years to integrate personalized, predictive, preventative, and participatory approaches in allergic diseases [133].

## 7. Summary of The Issues of Asthma

Throughout this review, we showed the various factors that play a role in the heterogeneity of asthma and the importance of understanding this variation to classify and better understand this disease. For example, asthma susceptibility risk loci have been identified to be related to immune cells; however, assessing the heritability of asthma has not been fully deciphered yet. Additionally, many environmental factors play a role in asthma development, with increased concerns over how different environmental exposure might trigger asthma exacerbations. Some of the complex gene-environment interactions that lead to asthma development include obesity, vitamin D deficiency, and the gut microbiome.

The diagnosis of asthma relies on several factors, making it challenging to properly diagnose asthmatic patients. Additionally, the correct diagnosis plays a role in managing this disease; however, choosing the correct treatment depends on the asthma endotype, clinical markers, and patient-focused aspects. The different asthma phenotypes can be characterized by the molecular and cellular events that drive the development and progression of asthma. Such events or biomarkers rely on systems biology and its various omics. The most common systems biology method used in asthma is transcriptomics, with many microarray datasets available in publicly available repositories. However, these datasets revealed extreme method-to-method variation due to the different experimental processes, which affects the identification of proper DEGs. Additionally, confounding factors appear to limit the benefits of transcriptomic studies on asthma, as these can create a bias in the experimental design. PBMCs were identified as a potential source of biomarkers as it includes many different allergic response mediators, and they are currently used to identify clinically essential biomarkers in diabetes [61].

## 8. Conclusions

To conclude, asthma is a heterogeneous disease of varying and overlapping phenotypes with many confounding factors in the disease susceptibility and management. The use of systems biology has helped us understand and enrich our knowledge of asthma heterogeneity and molecular basis; however, such methods have their limitations. Thus, the translational benefits of these studies are few, and it is recommended to reanalyze the different studies and omics in conjugation with one another to understand the reasons for this variation, which might help overcome the limitations of understanding the heterogeneity in asthma pathology.

## Figures and Tables

**Table 1:** Different subtypes of persistent bacterial bronchitis.

Subtypes	Definition
PBB—micro	History of chronic cough; positive BAL cultures; 2-week amoxicillin–clavulanic acid course
PBB—clinical	History of chronic cough; 2-week amoxicillin–clavulanic acid course
PBB—extended	PBB-micro or PBB extended; 4-week antibiotic course
PBB—recuring	More than 3 attacks of PBB-micro or PBB-clinical annually

PBB = persistent bacterial bronchitis; BAL = bronchoalveolar.
